# Potent antiviral activity of carbohydrate-specific algal and leguminous lectins from the Brazilian biodiversity†
†Electronic supplementary information (ESI) available. See DOI: 10.1039/c8md00508g


**DOI:** 10.1039/c8md00508g

**Published:** 2019-01-14

**Authors:** Ana C. S. Gondim, Suzete Roberta da Silva, Leen Mathys, Sam Noppen, Sandra Liekens, Alexandre Holanda Sampaio, Celso S. Nagano, Cintia Renata Costa Rocha, Kyria S. Nascimento, Benildo S. Cavada, Peter J. Sadler, Jan Balzarini

**Affiliations:** a Department of Biochemistry and Molecular Biology , Federal University of Ceará , 60455-760 , Fortaleza , Ceará , Brazil . Email: bscavada@gmail.com; b Department of Chemistry , University of Warwick , Coventry CV4 7AL , UK . Email: P.J.Sadler@warwick.ac.uk; c Department of Organic and Inorganic Chemistry , Federal University of Ceará , 60455-900 , Fortaleza , Ceará , Brazil; d Department of Fishing and Engineering , Federal University of Ceará , 60455-900 , Fortaleza , Ceará , Brazil; e Rega Institute for Medical Research , Department of Microbiology and Immunology , KU Leuven , 3000 Leuven , Belgium . Email: jan.balzarini@kuleuven.be; f Biochemistry Department , LINKA/UFPE , CEP 50670-901 , Recife , Pernambuco , Brazil; g Para West Federal University , 68220-000 , Monte Alegre , Brazil

## Abstract

Brazil has one of the largest biodiversities in the world. The search for new natural products extracted from the Brazilian flora may lead to the discovery of novel drugs with potential to treat infectious and other diseases. Here, we have investigated 9 lectins extracted and purified from the Northeastern Brazilian flora, from both leguminous species: *Canavalia brasiliensis* (ConBr), *C. maritima* (ConM), *Dioclea lasiocarpa* (DLasiL) and *D. sclerocarpa* (DSclerL), and algae *Amansia multifida* (AML), *Bryothamniom seaforthii* (BSL), *Hypnea musciformis* (HML), *Meristiella echinocarpa* (MEL) and *Solieria filiformis* (SfL). They were exposed to a panel of 18 different viruses, including HIV and influenza viruses. Several lectins showed highly potent antiviral activity, often within the low nanomolar range. DSclerL and DLasiL exhibited EC_50_ values (effective concentration of lectin required to inhibit virus-induced cytopathicity by 50%) of 9 nM to 46 nM for HIV-1 and respiratory syncytial virus (RSV), respectively, DLasiL also inhibited feline corona virus at an EC_50_ of 5 nM, and DSclerL, ConBr and ConM showed remarkably low EC_50_ values ranging from 0.4 to 6 nM against influenza A virus strain H3N2 and influenza B virus. For HIV, evidence pointed to the blockage of entry of the virus into its target cells as the underlying mechanism of antiviral action of these lectins. Overall, the most promising lectins based on their EC_50_ values were DLasiL, DSclerL, ConBr, ConM, SfL and HML. These novel findings indicate that lectins from the Brazilian flora may provide novel antiviral compounds with therapeutic potential.

## Abstract 2

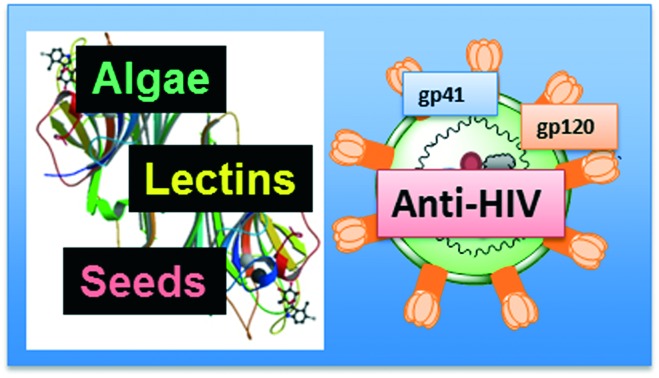
Brazilian legumes and algae contain potent antiviral lectins.

## Introduction

For decades, lectins have been recognized as potential natural drugs capable of treating a range of diseases, and hence their biological activity has been studied intensely.^1^–^5^ In this context, Brazilian biodiversity can potentially provide a wealth of novel unknown lectins, which can be mined for novel bioactivity.

Lectins are found in a broad range of organisms, from land plants to marine algae, and are capable of discriminating between, and binding to specific carbohydrate structures.^6^ The location of the carbohydrate binding sites on the lectin, sugar-binding epitopes and ligand disposition on its scaffold, give rise to a variety of types of interactions of lectins with their sugar ligands.^7^ The avidity and specificity for binding to glycoconjugates relies on the carbohydrate recognition domains (CRD) of the lectin on the one hand, and the density of the glycans, the glycan structure, and their multivalency, on the other.^8^

Recently the therapeutic potential of lectins from algae has been widely recognized.^9^ Algae are indeed a rich natural marine resource of lectins on the Brazilian coast that may be endowed with important pharmacological activities. For example, lectins from *Bryothamnion triquetrum* (BTL) and *Bryothamnion seaforthii* (BSL) have been used to differentiate human colon carcinoma cell variants,^10^ while the algal (cyanobacterial) lectin cyanovirin-N (CV-N) shows not only potent antiviral activity against HIV-1 and HIV-2, but also against influenza virus and Ebola virus.^7^

Lectins are proteins found in nature. This can have several disadvantages, including expensive production and scaling-up, poor oral bioavailability, hemagglutination of human red blood cells, mitogenic activity, cellular toxicity and stimulation of differentiation/activation markers.^11^,^12^ The cyanobacterial lectin cyanovirin is a well-known example for several of these properties.^13^ However, these potential compromising properties are highly dependent on the exact nature of the particular lectin. For example, many lectins agglutinate red blood cells derived from several animal species such as mice, rats, rabbits, but not of human origin.^11^,^14^ Microvirin, an alpha(1,2)-mannose-specific lectin from *Microcystis aeruginosa* has been shown to have comparable anti-HIV activity as cyanovirin, but a much higher safety profile. It proved 50-fold less cytotoxic than cyanovirin, and it did not increase the level of activation markers in CD4^+^ T lymphocytes.^15^ Also, the highly potent antiviral red algal lectin griffithsin was reported to have an outstanding safety and efficacy profile as a potential microbicidal drug candidate.^16^ It induces only minimal changes in secretion of inflammatory cytokines and chemokines by epithelial cells and human PBMCs, does not markedly upregulate T cell activation markers and gene expression, and has no measurable effect on cell viability. Even in the case of the mitogenic activity of a particular lectin such as the banana lectin BanLec, it was recently shown that its mitogenic activity could be uncoupled from its antiviral activity by engineering this lectin through site-directed mutagenesis. Thus, lectins can be modulated to remove a non-desirable activity (*i.e.* mitogenicity) while preserving a beneficial activity (*i.e.* antiviral activity).^17^ Moreover, since carbohydrate-binding agents such as lectins are prime candidate drugs for preventing sexually-transmitted viral infections (*i.e.* HIV, HBV, HCV, herpes viruses), oral bioavailability of such drugs is not required, and it can even be an advantage to have poor absorption through cell layers in order to avoid undesired systemic side effects.^18^ Evidence that lectins can realistically be used to treat pathogen infections *in vivo* has recently been provided by several investigators. Scalable manufacture of cyanovirin^19^ and griffithsin^20^ and validation of the safety and antiviral efficacy of griffithsin in mice and rabbits as a topical microbicide has been reported by O'Keefe *et al.*,^21^,^22^ Takebe *et al.*,^23^ Kouokam *et al.*,^24^ Farr Zuend *et al.*^25^ and Girard *et al.*^26^ Interestingly, Castillo-Acosta *et al.*^27^ recently demonstrated that Pradimicin, a mannose-specific agent purified from *Actinomyces madura*, can cure mice suffering from acute sleeping sickness caused by trypanosomes.

Currently, legume lectins are the most extensively studied, including lectins from seeds of plants belonging to the Fabaceae family.^28^*Dolichos lablab* lectin (DLL) has been shown to weaken proangiogenic signals, specifically nuclear factor kappa B (NF-κB), matrix metalloproteinase (MMP-2 and 9) and vascular endothelial growth factor (VEGF) in lectin-exposed mice.^29^*Lens culinaris* agglutinin and *Pisum sativum* agglutinin are capable of disrupting HIV infection by preventing the interaction of viral surface glycoprotein gp120 with the cellular CD4 receptor.^28^

Here, we show that several algal and leguminous lectins from the Brazilian flora are highly potent inhibitors towards a variety of viruses, and discuss their target sites and possible mechanisms of action.

## Results

The purified algal and leguminous lectins were initially investigated for their inhibitory activity against two different strains of HIV, HIV-1 (NL4.3) and HIV-2 (ROD). For this assay, HIV-1 and HIV-2 infection was performed in human CD4^+^ T-lymphocyte (CEM) cell cultures. The EC_50_ values for legume lectins (ConBr, ConM, DLasiL and DsclerL) were found to be in the nanomolar range and were generally somewhat lower (higher activity) for HIV-1 than HIV-2 (2- to 4-fold). Interestingly, DLasiL and DSclerL from Diocleinae species were up to 3-fold more active than the lectins derived from Canavalia species (Table 1).

**Table 1:** Antiviral activity of lectins against HIV-1 and HIV-2 infection of CD4^+^ CEM lymphocyte cell cultures and against syncytium formation between uninfected CD4^+^ SupT1 and persistently HIV-1-infected HUT-78/HIV-1 cells

Lectin	EC_50_^*a*^ (nM)			CC_50_^*b*^ (nM)
	CEM T-lymphocyte cultures		SupT1 + HUT-78/HIV-1 co-cultures	CEM
	HIV-1	HIV-2		
Leguminous lectins				
ConBr	73	137	75	100 ± 10
ConM	65	108	75	89 ± 4
DLasiL	31	89	46	>1000
DSclerL	20	88	63	220 ± 0
HHA	6.0 ± 2.0	3.6 ± 2.2	25.0 ± 7.0	>1000
Algal lectins				
AML	775	2079	1550	—
BSL	4521	>11 027	5293	233 ± 16
HML	60	>100	>100	—
MEL	>3333	>3333	—	—
SfL	440	304	326	—

^*a*^50% – effective concentration or compound concentration required to inhibit giant cell formation by 50%.

^*b*^50% – cytostatic concentration or compound concentration required to inhibit CEM cell proliferation by 50%.

In a second series of anti-HIV assays, co-cultivation of uninfected SupT1 and persistently HIV-1-infected HUT-78 cells (expressing the viral surface glycoproteins gp120 and gp41) was performed. In these assays, giant cells (syncytia) derived from the fusion between the infected and the uninfected cells (by virtue of gp120/gp41-CD4 interaction) were abundantly formed within 20 hours of co-cultivation. The EC_50_ values to prevent giant cell formation in the co-cultures in the presence of different concentrations of the lectins proved to be quite similar to the range of EC_50_ values for HIV-1 infection in the previously mentioned set (Table 1). These results indicate that leguminous lectins most likely block the adsorption/entry of the virus in the infection step, presumably by binding to the heavily glycosylated gp120/gp41 that is expressed on persistently HIV-1-infected HUT78/HIV1 cells. The antiviral activity concentrations of the leguminous lectins were usually well below their toxicity threshold (Table 1).

The algal lectins (HML, BSL, AML, MEL) were much less effective in blocking HIV-1 and HIV-2 activity than the leguminous lectins. For example, whereas HML was 3-fold less effective than the most active DSclerL lectin against HIV-1, SfL was 22-fold less inhibitory in comparison to the leguminous DSclerL lectin with EC_50_ values of 440 and 304 nM against HIV-1 and HIV-2, respectively. These remarkable differences between algae and leguminous lectins illustrate the different selective recognition properties of the lectins, which should be further explored.

Given the potent anti-HIV activity of several lectins, and their pronounced effect on syncytium formation in SupT1–HUT-78/HIV-1 co-cultures, the binding of the lectins to the HIV-1 envelope glycoproteins gp120 and gp41 and the cellular CD4 receptor was investigated by surface plasmon resonance (SPR) technology. These preliminary SPR studies suggest that HIV-encoded surface glycoproteins are potential antiviral targets for the lectins. No attempt was made to determine binding constants (*K*_D_) and related association (*k*_a_) and dissociation (*k*_d_) rates since the interpretation of data is complicated by the known auto-proteolysis of the leguminous lectins (Fig. S1†). The glycoproteins were bound on the sensor chip surface and binding of lectins flowing over the surface was recorded. The mannose-specific red algae-derived griffithsin lectin was used as a positive control for the assay. We selected only those lectins with promising EC_50_ values against HIV-1 including DLasiL, DSclerL, ConBr, ConM, SfL and HML. The leguminous lectins (DLasiL, DSclerL, ConBr, ConM) appeared to show higher binding to the three glycoproteins than the algal (SfL, HML) lectins (Table S1†). Also, the lectins appeared to bind to a higher extent to gp120 and gp41. Interestingly, there appears to be a positive correlation between the antiviral potential of the lectins in both primary infection and co-cultivation assays, and the binding of the lectins to gp120, gp41 and CD4. These preliminary SPR studies suggest that HIV-encoded surface glycoproteins are potential antiviral targets for the lectins and that future detailed measurements on a low density ligand sensor chip to determine *K*_D_, *k*_a_ and *k*_d_ values would be worthwhile, if the problems of auto-proteolysis and glycation can be controlled.

Since, besides HIV, also influenza viruses contain a heavily glycosylated envelope, three influenza viruses, influenza A (H1N1 subtype), influenza A (H3N2 subtype) and influenza B were investigated using a cytopathic evaluation and a coloured dye-MTS exposure assay in the presence of each of all 9 lectins. Remarkably, the leguminous lectin DSclerL showed exquisitely potent antiviral activity against influenza A (H3N2) infection with an EC_50_ as low as 400–1200 pM (Table 2). It proved to be 2- to 4-fold more active than ConBr. In contrast, much lower (if any) activity was observed towards the influenza A H1N1 strain (EC_50_ >20 000 pM). The three leguminous lectins were also markedly active against influenza B virus (EC_50_ in the higher picomolar/lower nanomolar range). Surprisingly, DLasiL, which was highly active against HIV, showed the poorest activity against all influenza virus strains. Despite a generally lower activity noticed for the algal lectins, some of them (*i.e.* AML) also showed nanomolar activity, although highly dependent on the nature of the influenza virus strain. The algae-derived SfL did not show anti-influenza virus activity at subtoxic (8–32 nM) concentrations. It proved to be markedly more cytotoxic than the other studied lectins. It is notable that the anti-influenza virus activity of the leguminous lectins was generally observed at concentrations that were markedly lower than their cytotoxic concentrations (40 to >200 nM).

**Table 2:** The inhibitory activity of lectins against influenza A (H1N1 and H3N2) and influenza B viruses

Lectin	EC_50_ (nM)						CC_50_ (nM)	MCC (nM)
	Influenza A (H1N1)		Influenza A (H3N2)		Influenza B			
	CPE^*a*^ (nM)	MTS (nM)	CPE (nM)	MTS (nM)	CPE (nM)	MTS (nM)		
Leguminous								
ConBr	>10	>10	2.1 ± 1	1.6 ± 1.5	2.0 ± 0.6	3.5 ± 3.7	>200	≥40
ConM	9 ± 7	9 ± 5	1.2 ± 0.5	0.23 ± 0.04	1.5 ± 1.9	0.50 ± 0.23	≥100	53.31
DLasiL	40 ± 0	12 ± 0	10 ± 2	8.0 ± 1.0	50 ± 20	42 ± 28	>200	>200
DSclerL	>20	>20	1.2 ± 1.2	0.4 ± 0.4	2.7 ± 0.9	6.0 ± 5	110 ± 68	86 ± 72
HHA	540 ± 360	—	2.0 ± 0.2	—	6.0 ± 2.0	—	—	>1000
Algal								
AML	105 ± 15	84 ± 3	25 ± 15	19 ± 7.5	12 ± 6	6.0 ± 3.0	≥300	≥300
BSL	>2000	>2000	220 ± 0	176 ± 33	600 ± 165	≥450	5900 ± 220	6600 ± 4400
HML	1400 ± 500	1010 ± 130	>2000	>2000	>2000	>2000	>10 000	>10 000
MEL	1100 ± 966	1266 ± 500	533 ± 200	530 ± 233	166 ± 66	82 ± 13	>3000	>3000
SfL	>6.0	>6.0	>6.0	>6.0	>6.0	>6.0	8	32

^*a*^Visual CPE score.

Finally, the activity of nine lectins against another set of 12 different viruses was determined, including Reovirus-1, Sindbis virus, Coxsackie B4 virus, Punta Toro virus, feline corona virus (FIPV), feline herpes virus, vesicular stomatitis virus, respiratory syncytial virus, herpes simplex virus 1 (KOS), herpes simplex virus 2 (G), herpes simplex virus 1 (TK-KOS ACV), vaccinia virus (Table 3). In these assays, the lectins did not show any promising activity towards most viruses such as reovirus-1, Sindbis virus, Coxsackie B4 virus, parainfluenza-3 virus, vaccinia virus and Punta Toro virus.

**Table 3:** Comparison of the inhibitory activity of lectins against viruses, different from HIV and influenza virus

Lectin	Virus EC_50_ (nM)												
	Para influenza 3-virus	Reovirus-1	Sindbis virus	Coxsackie virus B4	Punta Toro virus	Feline corona virus	Feline herpes virus	Vesicular stomatitis virus	Respiratory syncytial virus	Herpes simplex virus-1 (KOS)	Herpes simplex virus-2 (G)	Vaccinia virus	Herpes simplex virus-1 TK-KOS
Legume													
ConBr	>196	>196	>196	>196	>196	78 ± 4	>196	200	130 ± 60	>39	>39	>39	>39
ConM	>196	>196	>196	>196	>196	18.5 ± 0.5	≥200	>98	33 ± 9	>39	>39	>39	>39
DLasiL	>197	>197	>197	>197	>197	5 ± 2	>200	>98	9 ± 0.9	>39	>39	>39	>39
DSclerL	>195	>195	>195	>195	>195	59 ± 45	>195	>39	46 ± 36	>39	>39	>39	>39
HHA	>2000	>2000	—	>2000	—	520 ± 12	220 ± 86	—	>2000	>2000	>2000	—	>2000
Algae													
AML	>3523	>3523	>3523	>3523	>3523	1163	1392	28 385	1233	>705	>705	>705	>705
BSL	>11 027	>11 027	>11 027	>11 027	>11 027	8171	1676	11 027	3473	>2205	>2205	>2205	>2205
HML	>10 010	>10 010	>10 010	>10 010	>10 010	3944	1942	10 010	>10 000	>2000	>2002	>2002	>2002
MEL	>3333	>3333	>3333	>3333	>3333	>3333	>3333	100	1933	>3333	>3333	>3333	>3333
SfL	>733	>733	>733	>733	>733	110	374	733	>733	>147	>147	>147	>147

## Discussion

Acquired immunodeficiency syndrome (AIDS) is caused by the human immunodeficiency virus (HIV), and the disease compromises the immune system, predominantly infecting T-cells, dendritic cells and macrophages.^30^ The World Health Organization (WHO) predicts that in the next few years more than 37 million people will be infected by HIV.^31^ HIV is an enveloped virus possessing a lipid bilayer protecting the viral RNA and capsid. At the surface of the virus, the glycosylated glycoproteins gp120 and gp41 are imbedded in, and part of, the viral envelope at the start of the infection. Glycoprotein gp120 binds to the CD4 receptor present on T-cells. CD4 binding induces changes in gp120 resulting in the exposure of envelope domains, which were previously hidden at the moment it binds to the host cells. Such uncovered domains may then bind to the CXCR4 or CCR5 co-receptors on the cell membrane. It is difficult for antibodies to reach the initially-uncovered surface protein domains efficiently, which also means that efficient vaccines are not available yet for HIV.^31^ The HIV surface glycoprotein gp120 is one of the most heavily glycosylated proteins known so far, and its molecular weight consists of ∼50% of glycans. Therefore, targeting these glycans by carbohydrate-binding agents (CBAs) has been proposed earlier as a potential therapeutic approach to suppress HIV.^32^ Indeed, due to their high specificity for binding to carbohydrate chains on cell surfaces, lectins are widely studied in the glycobiology field, and participate in many important physiological processes.^31^ Several lectins have been reported as antiviral agents, but relatively few types of viruses have been investigated so far.^9^ The best-studied cases of lectins with potent antiviral (*i.e.* HIV) activity have been the cyanobacterial cyanovirin N algal lectin and the red algal lectin griffithsin, which have picomolar potency against HIV-1.^33^ Interestingly, several lectins with anti-HIV activity show recognition of different carbohydrates. For example, *Polygonatum citronella* and *Ophiopogon japonicus* lectins have affinity for sialic acid.^31^,^34^ Griffithsin binds to mannose/glucose structures, cyanovirin N predominantly binds to alpha(1,2)-mannose structures, *Galanthus nivalis* agglutinin (GNA) and *Hippeastrum* hybrid agglutinin exhibit α(1,3)/α(1,6)-mannose-binding activity^33^ and *Musa acuminata* agglutinin (BanLec) binds to high mannose carbohydrate structures.^35^ The latter lectins showed different degrees of anti-viral activity in the high picomolar range (*i.e.* griffithsin) or low nanomolar range.^33^ They block viral entry as the major mechanism of antiviral activity.

Thus, these and other lectins might act as antiviral compounds efficiently preventing viral entry into the host cells, which generally occurs through specific interactions of the lectins with glycans exposed on the gp120 (and gp41) glycoproteins (in the case of HIV) of the virus surface.^31^ In fact, the first lectin discovered to have anti-HIV activity, ConA, binds to glycoprotein structures, specifically at α-d-mannosyl and α-d-glucosyl groups found in the glycoproteins gp120 of HIV-1 and HIV-2,^31^,^36^ and blocks the binding of HIV to its receptors on the host cells.^37^ ConBr, ConM, DLasiL and DSclerL reported here have the same specificity and high identity of amino acid sequence with ConA (>97% for lectins from Canavalia and up to 80% for Dioclea lectins). Differences in their amino acid sequence result in distinct biological activities, as noticed in our study.^38^ Our data for HIV show antiviral potency from 20 to 137 nM for the leguminous lectins, while algal lectins tested were generally less potent (Table 1).

Algae lectins are structurally classified into families and according to their binding profile.^39^ In our study, AML, MEL and SfL belong to the OAAH lectin family that includes lectins from marine red algae *Eucheuma serra* (ESA) and *Kappaphycus alvarezii* (KAA) and cyanobacteria *Oscillatoria agardhii* (OAA). These lectins inhibited HIV infection through binding to high mannose (HM) oligosaccharides of enveloped glycoprotein gp120.^40^ These carbohydrate profiles indicate that this family has anti-HIV activity preferring to link α1-3 Man branched from the α1-6 Man of the penta-saccharide core, showing high affinity to HM *N*-glycans.^40^ OAA recognizes major high mannose sugars on HIV-1 showing similar EC_50_ values supporting the similarities of the family.^40^ This supports our findings for the algal lectins AML, MEL and SfL, where EC_50_ values showed minor differences that are associated with the number of sugar-binding epitope sites on the glycans that influence the anti-HIV activity.

The reason why the leguminous lectins have superior antiviral potential compared to the algae-derived lectins studied here may not only be due to their different specificities for sugar structures but also due to their tetrameric structure compared to the monomeric conformation of the algae-derived lectins (Table 4).

**Table 4:** Properties of the lectins studied here

Lectin	Carbohydrate/glycoprotein specificity	MW^*a*^ (kDa)	Structure	Ref.
Leguminous lectin				
1. ConBr	Glucose/mannose	25.5	Tetramer	41
2. ConM	Glucose/mannose	25.4	Tetramer	42
4. DLasiL	Glucose/mannose	25.4	Tetramer	43
3. DSclerL	Glucose/mannose	25.6	Tetramer	44
Algal lectin				
5. AML	Avidin, fetuin, yeast mannan	∼30	Monomer	45
6. BSL	Fetuin, mucin and avidina	9.0	Monomer	46, 47
7. HML	Thyroglobulin, porcine and bovine mucin	9.3	Monomer	48
8. MEL	Yeast mannan	28.9	Monomer	49
9. SFL	Yeast mannan	27.5	Monomer	50, 51

^*a*^Molecular weight of the subunit.

The tetrameric lectins (as also the case for *Hippeastrum* hybrid (Amaryllis) HHA and *Galanthus nivalis* (GNA)) have multiple carbohydrate-binding sites allowing tighter binding to, and cross-linking of the glycans on the viral envelope.^52^,^53^

It would be of interest to obtain structural data on the molecular interactions of the most active lectins in complexes with their HIV gp120 target. Generating NMR data, X-ray based crystallographic analysis and/or cryo-EM data will be challenging, but would indeed add significantly to understanding the molecular interactions of the lectins with the glycans on HIV gp120 and will be important for the rational design of mutated lectins with improved interactions with their targets and eventual antiviral efficacy.

Indeed, it has been shown earlier that lectins with preferential alpha-1,2; 1,3 and 1,6-mannose specificity usually show potent antiviral (HIV) activity,^14^,^18^,^32^,^54^ due to the presence of a number of clustered high-mannose-type glycans on the surface glycoprotein of HIV.^32^ Also, it can be assumed that tetrameric lectins may have a higher degree of interaction with their target glycans than monomeric lectins. As a result, they may have a higher capacity to cross-link the glycans on HIV gp120/gp41 thereby “freezing” the glycans in a fixed conformational state and decreasing the overall flexibility of the surface glycoprotein.

The lectins showed pronounced and comparable inhibition of both primary HIV infection (4 day assay) and giant cell formation in co-cultures of persistently HIV-1-infected and uninfected T-lymphocyte cells (∼20 h assay). These findings are highly suggestive for virus adsorption/entry as the underlying mechanism of antiviral action. The binding of the lectins to HIV-1 gp120 (and gp41), as measured by SPR, and the marked positive correlation between binding of gp120 (and gp41) and antiviral activity strongly corroborate this hypothesis. The fact that the lectins also measurably bind to CD4 might explain their strong overall anti-HIV activity, but may make these lectins somewhat less selective than griffithsin (that strongly binds to gp120/gp41, but interacts poorly with CD4). Further studies should reveal whether the additional binding of the lectins to CD4 eventually turns-out to be an advantage (in terms of antiviral efficacy), or a disadvantage (in terms of potential side-effects).

Another interesting set of viruses consists of the human influenza virus strains A and B. In particular the influenza A strains are very susceptible to antigenic drift and/or shift. They are subdivided based on the type of envelope substructures, *e.g.* influenza virus type A consisting of a well-defined hemagglutinin (HA) and neuraminidase (NA), which can be subdivided into 18 (H1–H18) and 11 (N1–N11) types.^55^ Influenza virus binds to sialic acid structures present on the host cell surface, through the viral envelope.^6^ According to the literature, humans and other vertebrates including wild birds, bats and pigs, can contract these type of viruses. Their infection can become pandemic and has been responsible for more than fifty million deaths. They can also cause highly destructive loss in domestic poultry and pose risks for humans.^6^,^55^ Lectin ESA-2 from red alga *Eucheuma serra* inhibits infection by influenza A H1N1, with an EC_50_ of 12 nM, by recognizing high mannose *N*-glycans (HM) on the HA surface glycoprotein.^40^ ConA also binds to HM glycans and showed a somewhat weaker activity with an EC_50_ of 41 nM, while lectins from *Aspergillus oryzae* (AOL) and *Aleuria aurantia* (AAL) that bind to fucose were less potent with EC_50_ values of 50 to 100 nM.^40^ Our results are quite remarkable, since we identified a leguminous lectin DSclerL, with exquisitely high activity against influenza A (H3N2) infection with an EC_50_ as low as 0.4–1.2 nM (Table 2). Also, ConM exhibited very low EC_50_ values (high activity) of 0.2 to 1.5 nM against influenza A (H3N2) and influenza B viruses. Beyond that, a high degree of selectivity for these lectins regarding their target influenza virus strain, was noticed, which, for ConM, showed about 50-fold higher activity toward influenza A (H3N2) than influenza A (H1N1) (Table 2). These striking differences in antiviral potential between different strains of influenza virus may most likely be due to (often subtle) differences in glycan structures and glycan content on the HA and/or NEU surface glycoproteins.

Most of the other viruses studied here have not been widely investigated and prominent studies on their interactions with lectins are lacking. However, some examples have been reported, such as the lectin from *Narcissus tazetta* (NTL) that shows an EC_50_ against respiratory syncytial virus of 88 nM,^56^ which is about 10-fold less potent than our findings for DLasiL against RSV (EC_50_ = 9 nM). Additionally, even the best activity against influenza B virus (EC_50_ = 8 nM) and influenza A H3N2 (EC_50_ = 51 nM) for NTL was also markedly lower than those found for DSclerL (H3N2, EC_50_ = 0.4–1.2 nM) and ConM (H3N2; B, EC_50_ = 1.6–3.5 nM) in our current study.

## Conclusions

The investigation of 9 lectins isolated from the Brazilian biodiversity flora against 18 different viruses that belong to a broad variety of viral families has revealed a remarkable panel of highly active lectins. Interestingly, the most active lectins were of leguminous origin, with activity reaching the picomolar level.

The pronounced antiviral potencies make them attractive as novel agents to be further investigated for their antiviral potential. They might be suitable for topical application. Our findings encourage the further investigation of the potential of these lectins as antiviral agents.

## Experimental

### Material and methods

#### Lectin purification

All the lectins used in this study have been well characterized by SDS-PAGE (Fig. S1 and S2†), mass spectrometry (MS) and primary structure determination as described in the literature, and showed similar levels of purity as those reported.

Lectins ConBr, ConM, DLasiL and DSclerL were purified by affinity chromatography on a Sephadex G50 column using a reported procedure.^1^,^4^,^5^ Lectins from *Amansia multifida* (AML), *Bryothamnion seaforthii* (BSL), *Hypnea musciformis* (HML), *Meristiella echinocarpa* (MEL) and *Solieria filiformis* (SfL) were purified by combination of ammonium sulphate precipitation and ion exchange chromatography on a DEAE-Sephacel column as previously described.^45^,^46^,^48^,^51^ Algae collections were authorized through our registration with SISBIO (Sistema de Autorização e Informação em Biodiversidade, ID: 33913-8) and SISGEN (Sistema Nacional de Gestão do Patrimônio Genético e do Conhecimento Tradicional Associado, ID: AC14AF9). All lectins analyzed by SDS-PAGE and stained with Coomassie blue showed a consistently high level of purity of at least 95%.

#### Antiviral assays

HIV-1 (NL4.3 strain) was obtained from the AIDS Research and Reference Reagent Program (Division of AIDS, NIAID, NIH) and the HIV-2 (ROD strain) from Prof. Luc Montagnier (at that time at the Pasteur Institute, Paris, France).

The first series of antiviral assays was performed against the laboratory HIV-1 (NL4.3) and HIV-2 (ROD) strains in human CD_4_^+^ T-lymphocyte CEM cell cultures. The cell culture infective dose (CCID_50_ being the virus dose which infected 50% of the number of cell cultures) was determined from the titration of the virus stocks in human T-lymphocyte CEM cell cultures. A dose of *ca.* 100 CCID_50_ was used to infect CEM cell cultures (3 × 10^5^ cells per mL) with HIV. A flat-bottomed microtiter tray was used to receive 100 μL of the virus-infected CEM cell suspension and 100 μL of the test compounds. The cultivation time was 4 days at 37 °C. Then, the formation of virus-induced giant cells was determined and quantified under the microscope.

The second series of antiviral experiments consisted of a co-cultivation assay whereby 5 × 10^4^ persistently HIV-1-infected HUT-78 cells (designated HUT-78/HIV-1) were mixed with 5 × 10^4^ uninfected CD_4_^+^ T-lymphocyte SupT1 cells in the presence of different concentrations of the test compounds. Syncytium formation in the co-cultures was determined and quantified after ∼20 h under the microscope. The determination of the EC_50_ was based on the compound concentration required to prevent syncytium formation by 50%.

All other antiviral assays (different from HIV) were based on inhibition of virus-induced cytopathicity in human embryonic lung (HEL) (using herpes simplex virus type 1 (HSV-1), HSV-2 (G), vaccinia virus and vesicular stomatitis virus), African green monkey (Vero) (using parainfluenza-3 virus, reovirus-1, Sindbis virus, Coxsackie B4 virus, and Punta Toro virus), human cervix carcinoma (HeLa) (using vesicular stomatitis virus, Coxsackie B4 virus, and respiratory syncytial virus), Madin–Darby canine kidney (MDCK) (using influenza A (H1N1 and H3N1) and influenza B virus) or Crandel feline kidney (CRFK) (using feline herpes virus; feline infectious peritonitis virus) cell cultures. Confluent cell cultures in microtiter 96-well plates were inoculated with 100 CCID_50_ of virus in the presence of varying concentrations of the investigated lectins (from 100 to 0.1 μg mL^–1^). Viral cytopathicity was recorded as soon as it reached completion in the control virus-infected cell cultures that were not treated with the lectins. The antiviral concentration was expressed as the EC_50_ or the effective concentration of lectin required to inhibit virus-induced cytopathicity by 50%.^3^

HEL, HeLa and MDCK cells were obtained from the American Type Culture Collection (ATCC), Manassas, Virginia, USA. CRFK cells were a kind gift from Prof. H. Egberink, University of Utrecht, Utrecht, The Netherlands.

#### Surface plasmon resonance measurements

The specific interactions of the lectins with immobilized HIV-1 gp120, HIV-1 gp41 and cellular CD4 receptor were investigated by surface plasmon resonance (SPR) technology using the biosensor Biacore T200 (GE Healthcare, Uppsala, Sweden). The lectins DLasiL, DSclerL, ConBr, ConM, SfL and HML were used at 100 nM in PBS (pH 7.4), except for DLasiL and HML that were also used at 500 nM, and injected for 2 min onto the gp120-, gp41-, CD4-bound surface of a CM5 sensor chip at a flow rate of 5 μL min^–1^ (association phase). Then, the change of the SPR response was monitored at 25 °C for another 8 min in the absence of the compound (dissociation phase). For immobilization, gp120, gp41 and CD4 were immobilized at 4690, 2300 and 5569 RU's in 10 mM sodium acetate buffer (pH 4.0).

## Definitions

ConBr: *Canavalia brasiliensis* lectin
ConM: *Canavalia maritima* lectin
DLasiL: *Dioclea lasiocarpa* lectin
DSclerL: *Dioclea sclerocarpa* lectin
AML: *Amansia multifida* lectin
BTL: *Bryothamnium triquetum* lectin
BSL: *Bryothamnium seaforthii* lectin
HML: *Hypnea musciformis* lectin
MEL: *Meristiella echinocarpa* lectin
SfL: *Solieria filiformis* lectin
HHA: *Hyppeastrum hybrid agglutinin*

## Conflicts of interest

The authors declare no competing financial interests.

## Supplementary Material

Supplementary information
