# Supplementary material for: Potent antiviral activity of carbohydrate-specific algal and leguminous lectins from the Brazilian biodiversity
Source: Medchemcomm. 2019 Jan 14;10(3):390–8. doi: 10.1039/c8md00508g (PMC6430086; doi:10.1039/c8md00508g)
Supplement: Supplementary file 1 [file MD-010-C8MD00508G-s001.pdf]

Electronic Supplementary Material (ESI) for MedChemComm.
This journal is © The Royal Society of Chemistry 2019

SUPPORTING INFORMATION

Potent antiviral activity of carbohydrate-specific algal and leguminous lectins from the Brazilian biodiversity

Ana C. S. Gondim[1,2,3#], Suzete Roberta da Silva[4,8], Leen Mathys[5,6], Sam Noppen[5], Sandra Liekens[5], Alexandre Holanda Sampaio[4], Celso S. Nagano[4], Cintia Renata Costa Rocha[7], Kyria S. Nascimento[1], Benildo S. Cavada[1], Peter J. Sadler[2*], Jan Balzarini[5*]*

1-Department of Biochemistry and Molecular Biology, Federal University of Ceará, 60455-760, Fortaleza, Ceará, Brazil.
2- Department of Chemistry, University of Warwick, Coventry CV4 7AL, UK
3- Department of Organic and Inorganic Chemistry, Federal University of Ceará, 60455-900, Fortaleza, Ceará, Brazil.
4- Department of Fishing and Engineering, Federal University of Ceará, 60455-900, Fortaleza, Ceará, Brazil.
5- Rega Institute for Medical Research, Department of Microbiology and Immunology, KU Leuven, 3000 Leuven, Belgium.
6- Current address: Janssen Pharmaceutica, 2340 Beerse, Belgium
7- Biochemistry Department, LINKA/UFPE, CEP 50670-901,
Recife, Pernambuco, Brazil.
8- Para West Federal University, 68220-000, Monte Alegre, Brazil.
# Current address: Department of Organic and Inorganic Chemistry, Federal University of Ceará, 60455-900, Fortaleza, Ceará, Brazil

Contents

Table S1. Surface plasmon resonance measurements

Figure S1. Denaturing polyacrylamide gel of leguminous lectins
Figure S2. Denaturing polyacrylamide gel of algal lectins

Surface plasmon resonance measurements

The binding of the lectins to the HIV-1 envelope glycoproteins gp120 and gp41 and the cellular CD4 receptor was investigated. The glycoproteins were bound on the sensor chip surface and binding of lectins flowing over the surface was recorded. The mannose-specific red algae-derived griffithsin lectin was used as a positive control for the assay. Griffithsin (200 nM), a well-known algae-derived potent anti-HIV lectin, showed comparable binding to gp120 and gp41 as DSclerL (~3,200 and ~ 2,850 RU, respectively). However, its binding to CD4 was much less pronounced (~ 100 RU *versus* 958 for DSclerL).

We selected only those lectins with promising EC_{50} values against HIV-1 including DLasiL, DSclerL, ConBr, ConM, SfL and HML. This approach allowed us to uncover a possible relationship between interaction intensity with gp120/gp41 and eventual antiviral activity. The sensor chips were loaded with a relatively high (RU) density of gp120, gp41 and CD4 to allow detection of weak lectin binding to the glycoproteins. Since the leguminous lectins are prone to auto-proteolysis we did not attempt to determine binding constants and related association and dissociation rates, since the observed SPR response may not be determined by a single peptide species. The data in Table S1 reveal interesting trends that may be related to activity.

Table S1. SPR data (RU values) after 2 min lectin exposure for selected lectins (100 nM) binding to gp120, gp41 and CD4.

Lectin	gp120	gp41	CD4
ConBr	1,017	1,213	128
ConM	1,827	2,044	338
DLasiL	4,586	4,095	1,186
DSclerL	3,570	3,115	958
SfL	775	383	270
HML	60	-	15

Characterisation of the lectins by gel chromatrography

Gel chromatography shows that the leguminous lectins are >95% pure and the algal lectins >98% pure.

Leguminous lectins

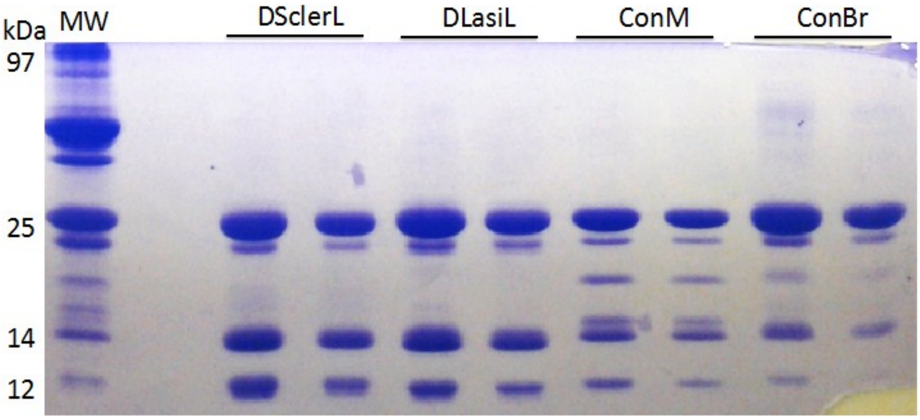

Figure S1. Denaturing polyacrylamide gel of leguminous lectins (loaded as 5 μL and 3 μL of protein sample per well).

Characterisation of the leguminous lectins on denaturing polyacrylamide gels gives band patterns typical of auto-proteolysis due to a post-translational processing (Figure S1), which is well known in the literature.[1-3] Smaller fragments derived of this spontaneous phenomenon are seen. These fragments are always produced and do not form active units.

For example, the mature *Canavalia brasiliensis* lectin (ConBr) monomer consists of a mixture of predominantly full-length polypeptide (α-chain) and a smaller proportion of fragments 1–118 (β-chain) and 119–237 (γ-chain).[4] *Canavalia Maritima* lectin (ConM) has a molecular mass of 102 kDa, by mass spectrometry and ultracentrifugation[5] when isolated by affinity cromatography on Sephadex G-50.[6] *Dioclea sclerocarpa* lectin shows molecular masses of full length α-chain (25,606 Da) and its derived fragments β-chain (12,832 Da), and γ-chain (12,752 Da), upon purification by affinity cromathograpy on Sephadex G-50.[7] *Dioclea lasiocarpa* lectin shows molecular masses of full length α-chain (25,410 Da) and its derived β-chain (12,816 Da), and γ-chain (12,611 Da), also upon purification by affinity cromathograpy on Sephadex G-50.[8] The four leguminous lectins that belong to a *Diocleinae* sub-tribe, show the same processing of proteolysis, and they have among themselves very high similarities in molecular sequences to ConA-like lectins. Also leguminous lectins are often glycosylated, which can explain the closely spaced bands (e.g. 24 and 25 kDa)

kDa)

The gels demonstrate at least 95% purity of the lectins on counting the bands all together, similar to previous reports in the literature.

Algal lectins

The algal lectins do not undergo auto-proteolysis processing, and they show high purity of >98%.

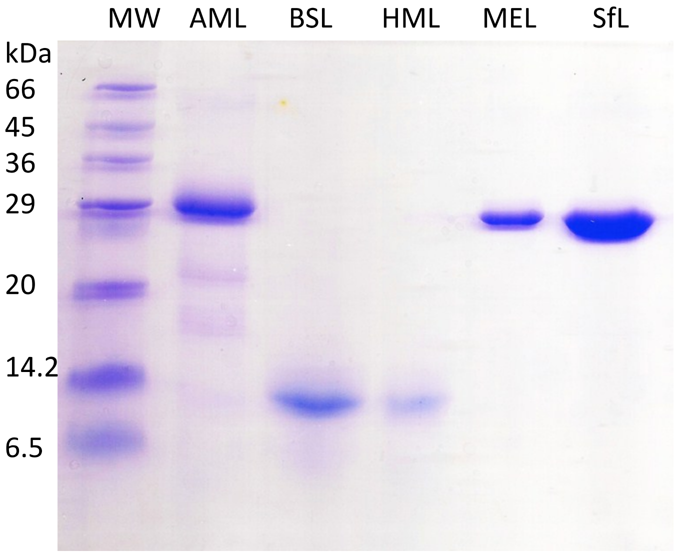

Figure S2. Denaturing polyacrylamide gel of algal lectins (AML[9], BSL[10], HML[11], MEL[12], SfL[13])

References

1. D. M. Carrington, A. Auffret and D. E. Hanke, *Nature*, 1985, **313**, 64-67.
2. J. J. Calvete, C. F. Santos, K. Mann, T. B. Grangeiro, M. Nimtz, C. Urbanke and B. Sousa-Cavada, *FEBS Lett.*, 1998, **425**, 286-292.
3. W. Min and D. H. Jones, *Nat. Struct. Biol.*, 1994, **1**, 502-504.
4. T. B. Grangeiro, A. Schriefer, J. J. Calvete, M. Raida, C. Urbanke, M. Barral-Netto and B. S. Cavada, *Eur. J. Biochem.*, 1997, **248**, 43-48.
5. P. Delatorre, B. A. Rocha, C. A. Gadelha, T. Santi-Gadelha, J. B. Cajazeiras, E. P. Souza, K. S. Nascimento, V. N. Freire, A. H. Sampaio, W. F. Azevedo, Jr. and B. S. Cavada, *J. Struct. Biol.*, 2006, **154**, 280-286.
6. M. V. Ramos, A. Moreira Rde, J. T. Oliveira, B. S. Cavada and P. Rouge, *Mem. Inst. Oswaldo Cruz*, 1996, **91**, 761-766.
7. J. L. Correia, A. S. do Nascimento, J. B. Cajazeiras, A. C. Gondim, R. I. Pereira, B. L. de Sousa, A. L. da Silva, W. Garcia, E. H. Teixeira, K. S. do Nascimento, B. A. da Rocha, C. S. Nagano, A. H. Sampaio and B. S. Cavada, *Molecules*, 2011, **16**, 9077-9089.
8. A. S. do Nascimento, A. C. Gondim, J. B. Cajazeiras, J. L. Correia, F. Pires Ade, K. S. do Nascimento, A. L. da Silva, C. S. Nagano, A. M. Assreuy and B. S. Cavada, *J. Mol. Recognit.*, 2012, **25**, 657-664.

9. S. A. Neves, A. L. Freitas, B. W. Sousa, M. L. Rocha, M. V. Correia, D. A. Sampaio and G. S. Viana, *Braz. J. Med. Biol. Res.*, 2007, **40**, 127-134.
10. L. G. do Nascimento-Neto, R. F. Carneiro, S. R. da Silva, B. R. da Silva, F. Vassiliepe Sousa Arruda, V. A. Carneiro, K. S. do Nascimento, S. Saker-Sampaio, V. A. da Silva, Jr., A. L. Porto, B. S. Cavada, A. H. Sampaio, E. H. Teixeira and C. S. Nagano, *Mar. Drugs*, 2012, **10**, 1936-1954.
11. C. S. Nagano, H. Debray, K. S. Nascimento, V. P. Pinto, B. S. Cavada, S. Saker-Sampaio, W. R. Farias, A. H. Sampaio and J. J. Calvete, *Protein Sci.*, 2005, **14**, 2167-2176.
12. R. P. Chaves, S. R. Silva, J. P. F. A. Silva, R. F. Carneiro, B. L. Sousa, J. O. Abreu, F. C. T. Carvalho, C. R. C. Rocha, W. R. L. Farias, O. V. Sousa, A. L. C. Silva, A. H. Sampaio, C. S. Nagano, *J. Appl. Phycol.*, 2018, DOI: 10.1007/s10811-018-1473-7.
13. R. P. Chaves, L. G. Nascimento Neto, R. F. Carneiro, A. L. C. D Silva, A. H. Sampaio, B. L. Sousa, M. G. Cabral, P. A. Videira, E. H. Teixeira, C. S. Nagano, *Int. J. Biol. Macromol.* 2018, **107**, 1320–1329 .
